# Allosteric Modulation of GCase Enhances Lysosomal Activity and Reduces ER Stress in GCase-Related Disorders

**DOI:** 10.3390/ijms26094392

**Published:** 2025-05-06

**Authors:** Ilaria Fregno, Natalia Pérez-Carmona, Mikhail Rudinskiy, Tatiana Soldà, Timothy J. Bergmann, Ana Ruano, Aida Delgado, Elena Cubero, Manolo Bellotto, Ana María García-Collazo, Maurizio Molinari

**Affiliations:** 1 Faculty of Biomedical Sciences, Institute for Research in Biomedicine, Università Della Svizzera Italiana, CH-6500 Bellinzona, Switzerland; ilaria.fregno@irb.usi.ch (I.F.); tatiana.solda@irb.usi.ch (T.S.); timothy.bergmann@irb.usi.ch (T.J.B.); 2 Gain Therapeutics, Sucursal en España, Parc Científic de Barcelona, 08028 Barcelona, Spain; nperez@gaintherapeutics.com (N.P.-C.); aruano@gaintherapeutics.com (A.R.); adelgado@gaintherapeutics.com (A.D.); ecubero@gaintherapeutics.com (E.C.); 3 Department of Biology, Swiss Federal Institute of Technology; CH-8093 Zurich, Switzerland; 4 GT Gain Therapeutics SA, CH-6900 Lugano, Switzerland; mbellotto@gaintherapeutics.com; 5 School of Life Sciences, École Polytechnique Fédérale de Lausanne, CH-1015 Lausanne, Switzerland

**Keywords:** Parkinson’s disease, Gaucher disease, glucosylceramidase beta 1 (GCase), lysosomal storage disorders, structurally targeted allosteric regulators (STARs), site-directed enzyme enhancement therapy (SEE-Tx), pharmacological chaperones, protein misfolding, lysosomal dysfunction, allosteric modulation

## Abstract

Variants in the *GBA1* gene, encoding the lysosomal enzyme glucosylceramidase beta 1 (GCase), are linked to Parkinson’s disease (PD) and Gaucher disease (GD). Heterozygous variants increase PD risk, while homozygous variants lead to GD, a lysosomal storage disorder. Some *GBA1* variants impair GCase maturation in the endoplasmic reticulum, blocking lysosomal transport and causing glucosylceramide accumulation, which disrupts lysosomal function. This study explores therapeutic strategies to address these dysfunctions. Using Site-directed Enzyme Enhancement Therapy (SEE-Tx^®^), two structurally targeted allosteric regulators (STARs), GT-02287 and GT-02329, were developed and tested in GD patient-derived fibroblasts with relevant GCase variants. Treatment with GT-02287 and GT-02329 improved the folding of mutant GCase, protected the GCase_Leu444Pro_ variant from degradation, and facilitated the delivery of active GCase to lysosomes. This enhanced lysosomal function and reduced cellular stress. These findings validate the STARs’ mechanism of action and highlight their therapeutic potential for GCase-related disorders, including GD, PD, and Dementia with Lewy Bodies.

## 1. Introduction

Glucocerebrosidase (GCase), encoded by the *GBA1* gene, is a lysosomal enzyme crucial for the hydrolysis of glucosylceramide into glucose and ceramide. Variants in *GBA1* that modify the protein’s sequence (e.g., G202R, N370S, D409H, and L444P) may affect the capacity of the polypeptide chain to attain the native structure and be delivered to the lysosomes [1,2,3]. Misfolded GCase proteins are typically retained within the endoplasmic reticulum (ER), where they are either subjected to ER-associated degradation (ERAD) pathways or accumulate, triggering ER stress responses [4]. The relationship between GCase dysfunction and such mechanisms has been extensively documented in early studies. Swakar et al. (2002) first highlighted the misfolding and ER retention of mutant GCase [5], a finding later confirmed in tissue culture systems by Schmitz et al. (2005) [6], Ron et al. (2005) [2], and Bendikov-Bar et al. (2011) [7]. Additionally, in vivo studies using Drosophila models demonstrated similar outcomes, as shown by Suzuki et al. (2013) [4], Maor et al. (2013) [8], and Sanchez-Martinez et al. (2016) [9].

Variants in the GCase polypeptide are associated with Gaucher disease (GD), a lysosomal storage disorder characterized by the accumulation of glucosylceramide due to impaired GCase activity. Notably, these variants also significantly increase the risk of developing Parkinson’s disease (PD) and Dementia with Lewy Bodies (DLB), making *GBA1* the most common genetic risk factor for PD [10,11]. These disorders are believed to involve a combination of lysosomal-autophagic pathway impairment, toxic substrate accumulation, and protein misfolding, which together exacerbate neurodegeneration.

Traditional therapeutic approaches for GCase-related disorders include enzyme replacement therapy (ERT) and substrate reduction therapy (SRT), which, while effective, have limitations such as high costs and potential side effects. Pharmacological chaperones (PCs), such as ambroxol and arimoclomol, represent alternative approaches. Ambroxol improves GCase activity, facilitates lysosomal transport, and reduces glucosylceramide buildup, as widely reported [9,12]. Similarly, arimoclomol enhances the heat shock response and has shown promise in preclinical models [13], highlighting its potential in GCase stabilization. However, both compounds mainly stabilize GCase without significantly boosting enzymatic function, underscoring the need for more effective therapies targeting both stability and activity.

**Figure 1:**
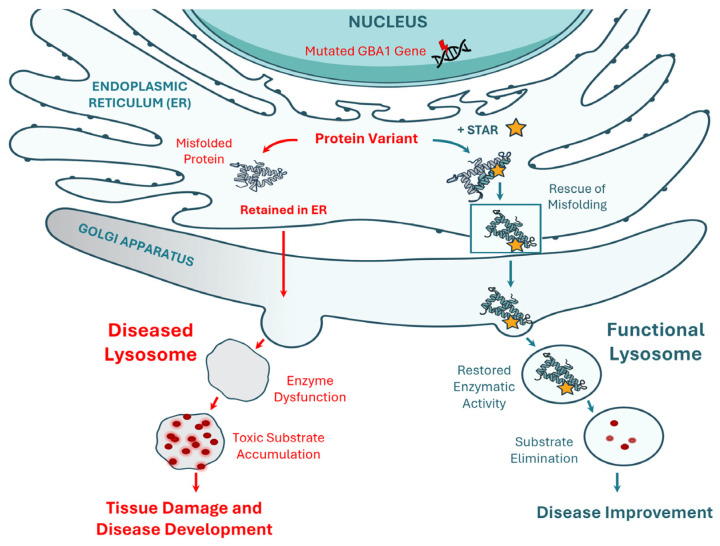
Targeting GCase offers a promising approach for disease-modifying treatment in Parkinson’s Disease. Gain Therapeutic’s structurally targeted allosteric regulators (STARs) address GCase misfolding, enhancing its enzymatic activity and promoting its transport to lysosomes. By restoring GCase function, these regulators provide neuroprotective effects, improve lysosomal health, and reduce toxic substrate buildup, thereby mitigating neurodegenerative processes. GCase, glucocerebrosidase. For a detailed view of the L444P and N370S variant locations relative to the active site, see Appendix A Figure A1.

Recent drug discoveries have introduced structurally targeted allosteric regulators (STARs) as a novel therapeutic strategy. STARs bind to allosteric sites on GCase [5], enhancing its activity without interfering with the enzyme’s catalytic site. As shown in Figure 1, this study evaluates GT-02287 and GT-02329, two STARs developed using the Site-directed Enzyme Enhancement Therapy (SEE-Tx^®^) platform [14,15,16,17]. These compounds bind to an allosteric site on GCase, enhancing its activity and facilitating its transport to lysosomes. By stabilizing mutant GCase variants, GT-02287 and GT-02329 reduce substrate accumulation and alleviate cellular stress associated with protein misfolding [18,19], offering a novel therapeutic approach [18,19] for GCase-related disorders, including GD, PD, and DLB.

## 2. Results

### 2.1. Introduction to GT-02287 and GT-02329

SEE-Tx^®^ technology [20,21,22] utilized the high-resolution structure of native human GCase (PDB: 2V3F) [23] to identify an allosteric binding site and evaluate its druggability. This process guided subsequent steps, where key binding hotspots informed a high-throughput virtual screening assay [24]. This approach led to the identification of novel virtual hits, which were subsequently purchased and tested for interaction with the GCase protein. The initial screening workflow has been detailed previously by Montpeyo et al., 2024 [17]. Experimentally validated hits from the screening step served as the foundation for a medicinal chemistry program, ultimately resulting in the identification of GT-02287 and GT-02329. Due to the proprietary nature of research in this drug development phase, the chemical structures of these compounds remain undisclosed.

### 2.2. GT-02287 and GT-02329 Bind to the GCase Protein in an Allosteric Pocket

GT-02287 and GT-02329 interact with GCase through an allosteric binding site, utilizing hydrogen bonds and hydrophobic interactions to stabilize the enzyme in its native conformation. This binding does not compete with the natural substrate, allowing GCase to maintain its catalytic activity. By stabilizing mutant GCase variants from the early stages of folding, these STARs enable them to evade cellular quality control mechanisms and reach the lysosomes, where they can perform their enzymatic function. This process enhances lysosomal activity and reduces toxic substrate accumulation, offering a therapeutic strategy for GCase-related disorders.

**Figure 2:**
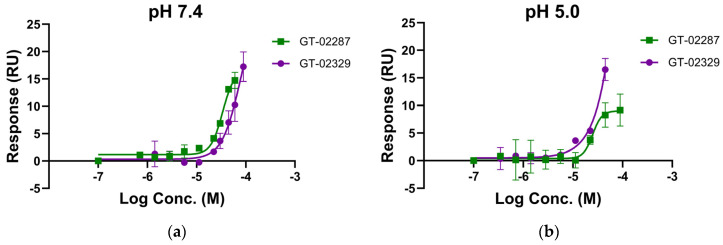
GCase binding confirmation: Surface plasmon resonance confirmed the direct binding of compounds GT-02287 and GT-02329 to recombinant human GCase protein (Cerezyme^®^) at (**a**) pH 7.4 and (**b**) pH 5.0. All values are presented as the mean ± SD from duplicate measurements. GCase, glucocerebrosidase; SD, standard deviation.

Surface plasmon resonance (SPR) confirmed the direct binding of GT-02287 and GT-02329 to recombinant human GCase protein (rhGCase) under neutral and acidic conditions. SPR enables the real-time measurement of molecular interaction kinetics and affinity, making it an excellent technique for this analysis [25]. Experiments were conducted at pH 7.4 and 5.0 to replicate the conditions of the ER and the acidic luminal environment of lysosomal compartments, respectively [26]. Both GT-02287 and GT-02329 exhibited dose-dependent binding to recombinant human GCase protein (rhGCase) under neutral and acidic conditions, with similar binding affinities observed at pH 7.4 and 5.0 (Figure 2a,b, respectively).

**Figure A2:**
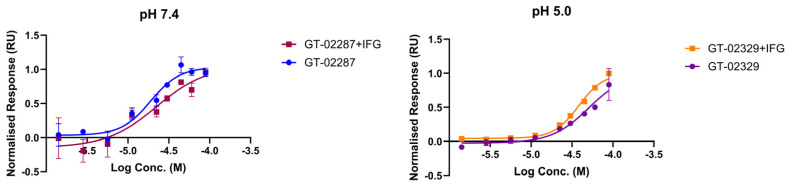
Surface plasmon resonance dose–response for GT compounds binding to immobilized recombinant human glucocerebrosidase (rhGCase) monitored at neutral (7.4) and acidic (5.0) pH with and without the presence of the inhibitory chaperone isofagomine (IFG).

SPR competition experiments with isofagomine (IFG), a competitive inhibitor of GCase, confirmed that GT-02287 and GT-02329 bind to an allosteric site rather than the active site of GCase. IFG binds to the active site of the GCase protein and acts as a PC, stabilizing mutant enzymes to improve their function. The dissociation constant (K_D_) values obtained with and without IFG were comparable at pH 7.4 and 5.0, indicating that these compounds bind to a pocket distinct from the active site occupied by IFG (Table 1; see Appendix A Figure A2).

Orthogonal biophysical experiments using nano-differential scanning fluorimetry (nanoDSF) [27] were performed to confirm the binding of GT-02287 and GT-02329 to rhGCase. The highly sensitive nanoDSF technique measures protein thermal stability by detecting changes in the intrinsic fluorescence of tryptophan and tyrosine residues during thermal unfolding. The thermal stabilization effects of GT-02287 and GT-02329 on rhGCase were evaluated at two concentrations (25 and 100 µM) alongside the positive IFG control.

At pH 7.0, both compounds significantly enhanced the thermal stability of rhGCase, with a difference in melting temperature (ΔTm) greater than 1 °C. They also showed a concentration-dependent increase in Tm compared to baseline, indicating effective protein stabilization and validating their interaction with GCase. The stabilizing effects were more pronounced at pH 7.0, where rhGCase is inherently less stable than at pH 5.0. At pH 5.0, GT-02287 showed no detectable effect on protein thermal stability, while GT-02329 and IFG both demonstrated stabilization. A detailed comparison of results at both pH levels is provided in Table 2, highlighting the pH-dependent efficacy of these compounds in stabilizing rhGCase.

### 2.3. GT-02287 and GT-02329 Increase GCase Activity in Fibroblast Lysates by Binding to a Site Distinct from the Active Site

**Figure 3:**
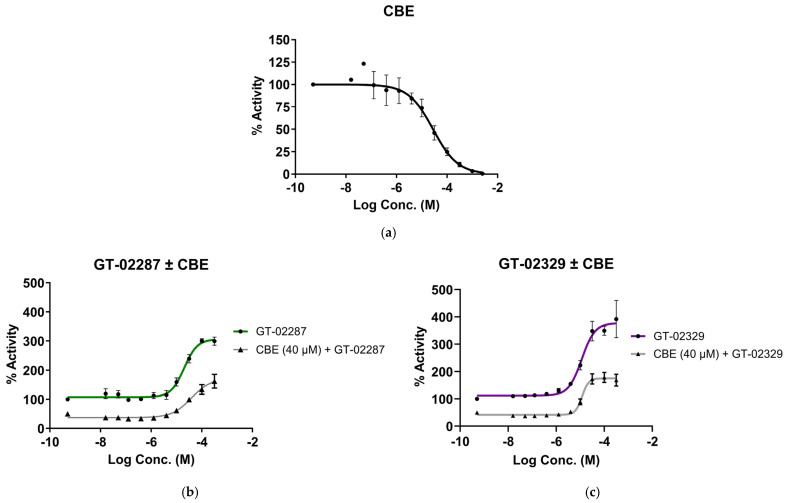
Competitive inhibition assay with CBE in lysates. Wild-type fibroblast lysates were incubated with (**a**) CBE (black); (**b**) GT-02287 (green); or (**c**) GT-02329 (purple) for 15 min at the indicated concentrations. GCase activity was measured using the 4-methylumbelliferyl β-D-glucopyranoside substrate. In (**b**) and (**c**), where indicated, an additional preincubation with 40 µM CBE (grey) was performed to inhibit 50% of GCase activity. All values represent the mean ± SD from three wells per condition. CBE, conduritol-β-epoxide; GCase, glucosylceramidase beta 1; SD, standard deviation.

The effect of GT-02287 and GT-02329 on GCase enzymatic activity was evaluated in wild-type fibroblast extracts at pH 5.6 (Figure 3a–c). Both compounds enhanced GCase activity at concentrations above 10 µM, as demonstrated using the substrate 4-methylumbelliferyl-β-D-glucopyranoside (Figure 3b,c). These findings suggest that the compounds bind to a site distinct from the enzyme’s catalytic site. If they bound to the active site, they would likely exhibit inhibitory behavior, like known inhibitors such as IFG [28,29].

Conduritol B epoxide (CBE), a potent and selective irreversible competitive inhibitor, covalently binds to the active site of GCase with an IC50 of 28.19 µM (Figure 3a) [30]. When the activity of GT-02287 and GT-02329 was tested in the presence of CBE at a concentration that irreversibly inhibits 50% of GCase activity, the enhancement of GCase activity by the GT compounds was reduced by 50% (Figure 3b and Figure 3c, respectively). This result confirms that the activity enhancement by GT-02287 and GT-02329 is mediated through their binding to GCase, demonstrating a clear relationship between their activity and the availability of functional GCase in fibroblast extracts.

### 2.4. GT-02287 and GT-02329 Enhance GCase Enzyme Activity in Primary Human Fibroblasts from Healthy Donors and GD Patients

**Figure 4:**
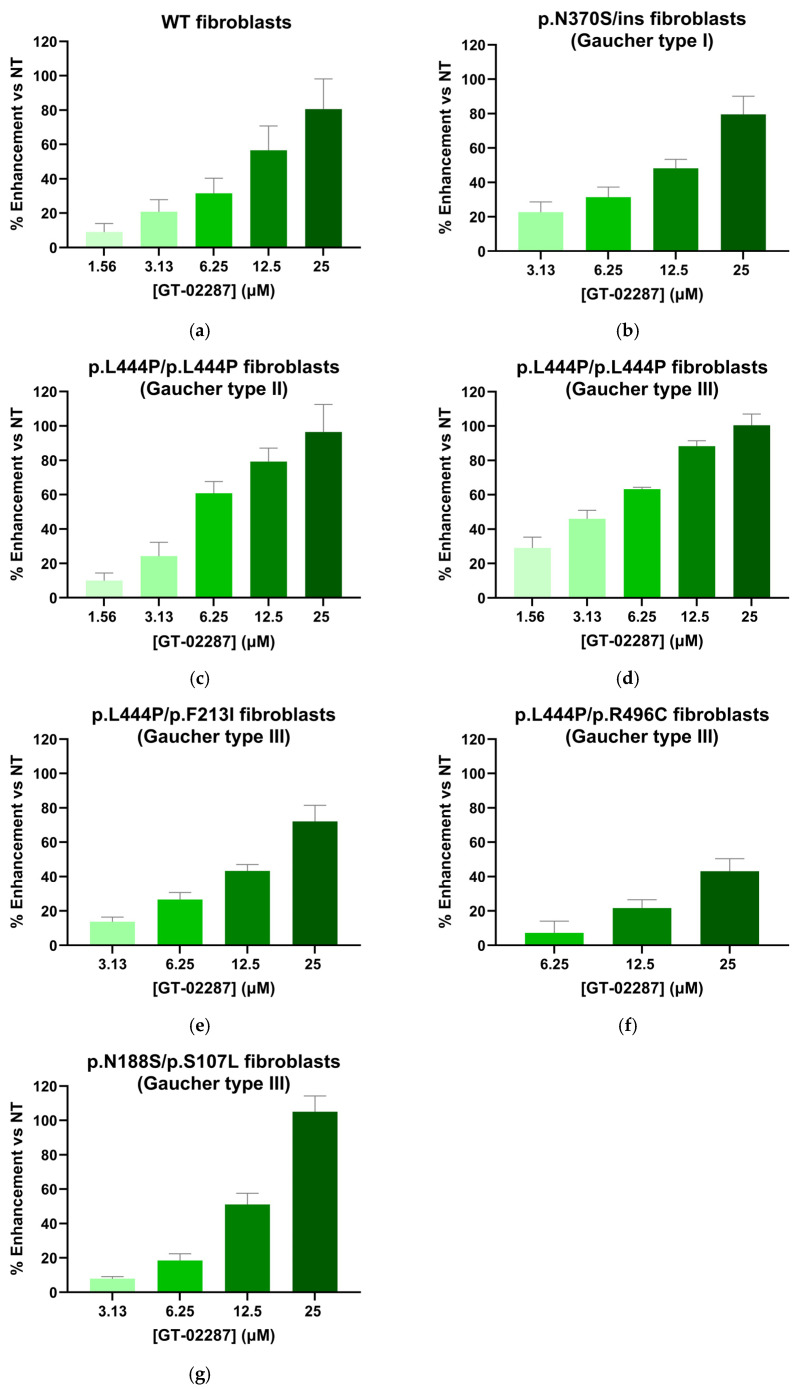
Cellular Gcase enhancing activity of GT-02287. Patient-derived fibroblasts (Coriell GM00372, Coriell GM08760, Telethon 20526, Telethon 21142, Telethon 20624, and Telethon 20843) were treated with GT-02287 (**a**–**g**) at the specified concentrations for 4 days. Gcase activity was measured using the fluorogenic substrate 4-methylumbelliferyl β-D-glucopyranoside. The dose–response effect is presented as the percentage increase in activity relative to untreated cells. Data represent the mean ± SD from at least three wells per condition. Gcase, glucocerebrosidase; SD, standard deviation.

**Figure 5:**
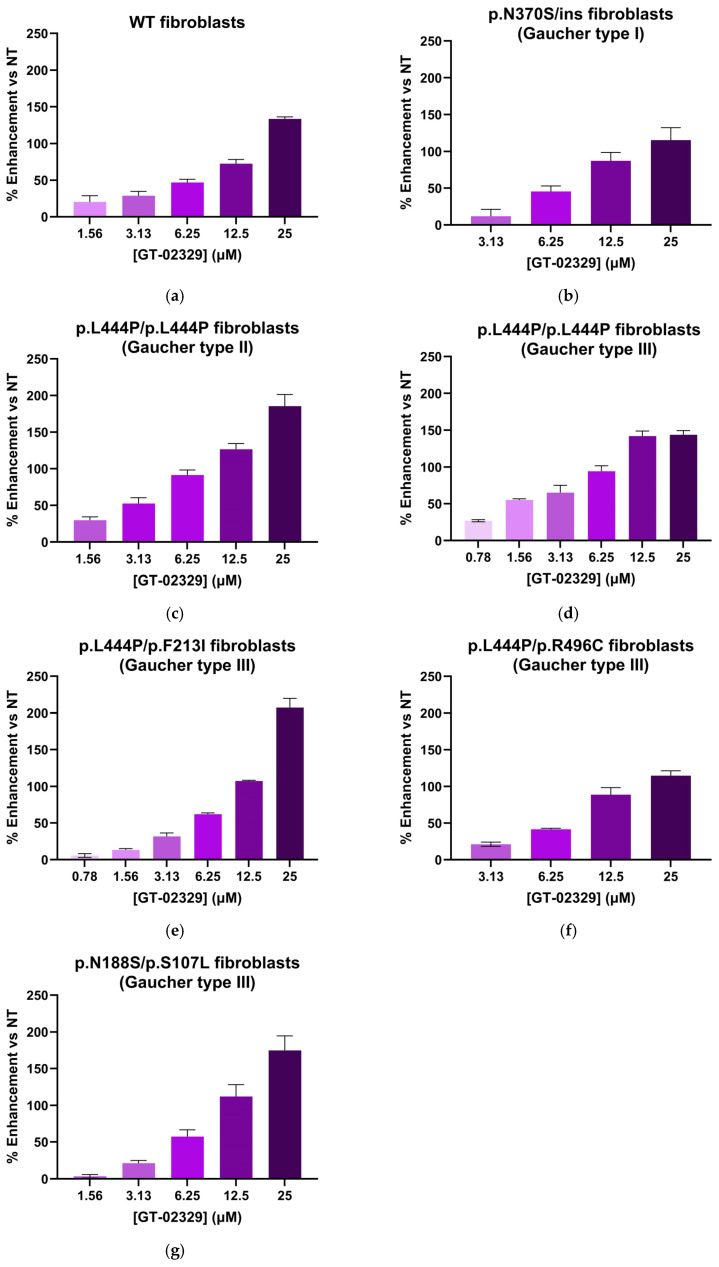
Cellular GCase enhancing activity of GT-02329. Patient-derived fibroblasts (Coriell GM00372, Coriell GM08760, Telethon 20526, Telethon 21142, Telethon 20624, and Telethon 20843) were treated with GT-02329 (**a**–**g**) at the specified concentrations for 4 days. GCase activity was measured using the fluorogenic substrate 4-methylumbelliferyl β-D-glucopyranoside. The dose–response effect is presented as the percentage increase in activity relative to untreated cells. Data represent the mean ± SD from at least three wells per condition. GCase, glucocerebrosidase; SD, standard deviation.

The ability of GT-02287 and GT-02329 to enhance the activity of GCase proteins with disease-causing variants was evaluated in primary human fibroblasts from a healthy donor (Figure 4a and Figure 5a, respectively) and six patients with GD types I, II, and III (Figure 4b–g and Figure 5b–g). The fibroblasts from these patients carried specific *GBA1* gene variants, including p.N370S/ins (GD type I); p.L444P/p.L444P (GD type II and III); p.L444P/p.F213I (GD type III); p.L444P/p.R496C (GD type III); and p.N188S/p.S107L (GD type III). In all cases, treatment with GT-02287 and GT-02329 resulted in a dose-dependent increase in GCase activity.

### 2.5. GT-02287 and GT-02329 Treatment Reduces Endogenous Substrate Levels in Primary Human Fibroblasts

**Figure 6:**
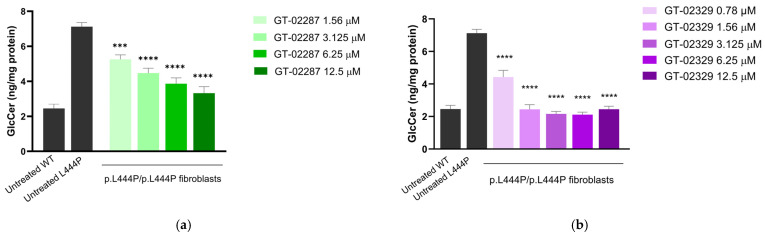
GlcCer substrate depletion following treatment with GT-02287 and GT-02329. p.L444P/p.L444P fibroblasts (Coriell GM08760) were treated with (**a**) GT-02287 or (**b**) GT-02329 at the specified concentrations for 10 days. Data represent the mean ± SD from three wells per condition. Statistical analysis was performed using one-way ANOVA followed by Dunnett’s Multiple Comparison Test. Asterisks indicate statistically significant differences compared to untreated GCase_Leu444Pro_ fibroblasts: *** *p* ≤ 0.001, **** *p* ≤ 0.0001. ANOVA, analysis of variance; GCase, glucocerebrosidase; GlcCer, glucosylceramide; SD, standard deviation.

Reduced GCase activity in patient-derived p.L444P/p.L444P primary fibroblasts results in an increase in glucosylceramide (GlcCer) levels, a primary physiological substrate of GCase, though this elevation differs from the pathological substrate accumulation observed in GD macrophages (Figure 6). To assess whether enhancing GCase activity with GT-02287 or GT-02329 reduces intracellular GlcCer levels, fibroblasts were treated for 10 days, starting one day after seeding. After treatment, cells were harvested and processed as described in the methods section. Quantification by ultra-high-performance liquid chromatography with tandem mass spectrometry (UHPLC-MS/MS) [31] demonstrated that treatment with increasing concentrations of GT-02287 (Figure 6a) or GT-02329 (Figure 6b) effectively reduced GlcCer levels in patient fibroblasts.

### 2.6. GT-02287 and GT-02329 Restore Lysosomal Transport of GCase

#### 2.6.1. Development of HaloTag Chimeras to Monitor Defective Lysosomal Transport of Disease-Causing GCase Variants by Confocal Laser Scanning Microscopy

To quantitatively assess the performance and mode of action of GT-02287 and GT-02329, a biochemical protocol was developed, building on methods previously used to evaluate STARs that enhance galactosidase beta 1 (GLB1) activity in GLB1-related LSDs such as GM1 gangliosidosis [16,18,19]. GCase and two disease-causing variants (GCase_Asn370Ser_ and GCase_Leu444Pro_) were tagged with a HaloTag (HT) polypeptide to create GCase-HT chimeras. HT allows fluorescent labeling using cell-permeable ligands like tetramethylrhodamine (TMR), enabling intracellular distribution analysis via confocal laser scanning microscopy (CLSM).

**Figure 7:**
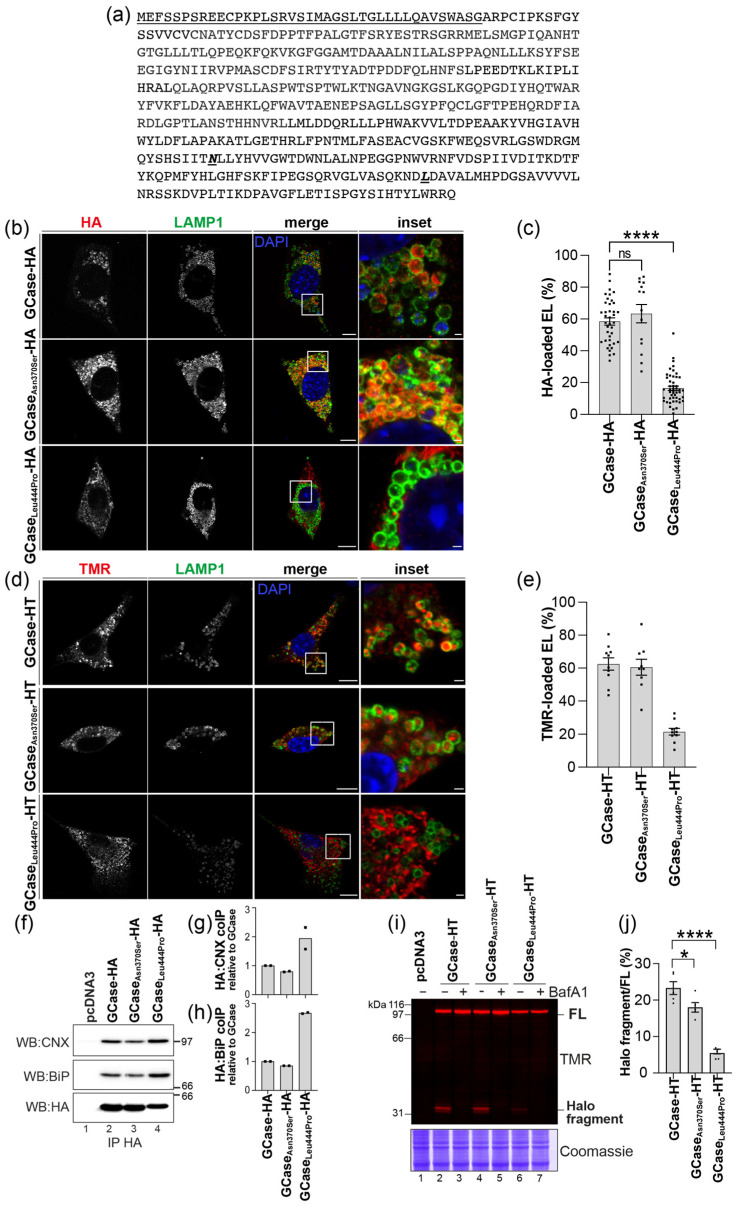
Lysosomal delivery of wild-type and disease-linked GCase variants: (**a**) sequence of GCase showing the signal sequence (underlined) and the disease-causing variants Asn370Ser and Leu444Pro (bold italics); (**b**) lysosomal delivery of HA-tagged GCase variants. Representative CLSM images of MEF cells expressing GCase variants for 48 h and treated with 50 nM BafA1 for 17 h. Immunostaining was performed with anti-HA (red) and anti-LAMP1 (green) antibodies, with nuclei stained using DAPI (blue). Scale bars: 10 µm (merged images) and 1 µm (insets); (**c**) quantification of (**b**) showing the percentage of LAMP1-positive lysosomes containing GCase variants. Images were analyzed using LysoQuant. Data are presented as mean ± SEM from three independent experiments. Statistical analysis: One-way ANOVA with Dunnett’s multiple comparison test; non-significant (ns) *p* > 0.05, **** *p* < 0.0001; (**d**) same as (**b**) for HT GCase variants. MEF cells were treated with 50 nM BafA1 and 100 nM TMR for 17 h. GCase variants were visualized with TMR (red), and lysosomes were stained with anti-LAMP1 (green). Scale bars: 10 µm (merged images) and 1 µm (insets); (**e**) quantification of (**d**) showing the percentage of LAMP1-positive lysosomes containing the TMR signal. Images were analyzed using LysoQuant. Data are presented as mean ± SEM from two independent experiments; (**f**) co-immunoprecipitation of GCase-HA variants in HEK293 cells. WB analysis shows CNX (upper panel), BiP (middle panel), and GCase-HA (lower panel); (**g**) quantification of CNX association with the three GCase variants; (**h**) quantification of BiP association with the three GCase variants. Data in (**g**) and (**h**) are presented as mean from two independent experiments; (**i**) fluorescent gel showing the TMR signal of GCase-HT variants expressed in HEK293 cells for 4 days, with or without 50 nM BafA1 treatment for the last 17 h; and (**j**) quantification of (**i**). Data are presented as mean ± SEM from five independent experiments. Statistical analysis: One-way ANOVA with Dunnett’s multiple comparison test; * *p* < 0.05, **** *p* < 0.0001. Bafilomycin A1, BafA1; binding immunoglobulin protein, BiP; calnexin, CNX; confocal laser scanning microscopy, CLSM; DAPI, 4′,6-diamidino-2-phenylindole; EL, endolysosomes; FL, full-length; GCase, glucocerebrosidase; HT, HaloTag; HA, hemagglutinin tag; HEK293, human embryonic kidney 293 cells; LAMP1, lysosomal-associated membrane protein 1; MEF, mouse embryonic fibroblasts; NT, non-transfected; SEM, standard error of the mean; TMR, tetramethylrhodamine; WB, Western blot.

Since HT (297 residues) is significantly larger than conventional tags like the human influenza hemagglutinin tag (HA; 9 residues) [31], their impact on the lysosomal delivery of GCase was evaluated (Figure 7a). GCase, GCase_Asn370Ser_, and GCase_Leu444Pro_ were tagged at their C-terminus with either HA or HT and transiently expressed in mouse embryonic fibroblasts (MEFs). Lysosomal delivery was assessed by immunostaining with anti-HA antibody (red) and anti-lysosomal-associated membrane protein 1 (LAMP1) antibody (green) by CLSM (Figure 7b) and quantified using LysoQuant [32] in cells treated with 50 nM bafilomycin A1 (BafA1). BafA1 inhibits lysosomal hydrolases and preserves material (the tagged versions of the GCase) delivered in the lumen of degradative LAMP1-positive endolysosomes (Figure 7c) [33]. Micrographs showed that GCase-HA (Figure 7b, upper panels) and GCase_Asn370Ser_-HA (Figure 7b, middle panels) were efficiently transported to LAMP1-positive lysosomes, while GCaseL_eu444Pro_-HA transport was highly defective (Figure 7b, lower panels and Figure 7c), correlating with the mild and severe GD phenotypes of these variants, respectively [10].

Similarly, MEFs transfected with HT variants and labeled with 100 nM TMR showed that GCase-HT and GCase_Asn370Ser_-HT were efficiently delivered to lysosomes, while GCase_Leu444Pro_-HT transport was defective (Figure 7d,e). Both HA and HT chimeras effectively reported on GCase transport efficiency from the ER to lysosomes.

Co-precipitation assays revealed that, unlike wild-type GCase and GCase_Asn370Ser_, the GCase_Leu444Pro_ variant exhibited stronger associations with ER chaperones calnexin (CNX) (Figure 7f, upper panel, lane 4, and quantification in Figure 7g) and binding immunoglobulin protein (BiP) (Figure 7f, middle panel, lane 4 and quantification in Figure 7h). This persistent association with ER-resident chaperones indicates the mutant GCase’s inability to efficiently attain its native conformation, leading to defective delivery to the endolysosomal compartment [34].

#### 2.6.2. HaloTag Chimeras to Monitor Defective Lysosomal Transport of Disease-Causing GCase Variants by Gel Electrophoresis

Upon delivery of chimeric proteins to lysosomal compartments, the HT is cleaved, generating acid- and protease-resistant fluorescent Halo fragments (~33 kDa). This allows for the direct visualization and quantification of lysosomal delivery by monitoring the formation of the 33 kDa fluorescent Halo fragment in gel electrophoresis [16,18,19,35,36,37,38].

Cells expressing GCase-HT, GCase_Asn370Ser_-HT, or GCase_Leu444Pro_-HT were grown in the presence of 100 nM TMR. After detergent solubilization, the post-nuclear supernatant (PNS) was separated by sodium dodecyl sulfate-polyacrylamide gel electrophoresis (SDS-PAGE), and TMR-labeled polypeptides were visualized using a 532 nm laser. The PNS of cells expressing GCase-HT contains two fluorescent polypeptides: the upper band (97 kDa) corresponds to full-length GCase-HT (Figure 7i, upper panel, lane 2, FL), while the lower band (33 kDa) represents the Halo fragment, generated upon lysosomal delivery where lysosomal enzymes cleave the linker between GCase and the HaloTag (Figure 7i, upper panel, lane 2, Halo fragment, and quantification in Figure 7j). The generation of the 33 kDa Halo fragment is blocked when cells are treated with 50 nM BafA1, which inactivates lysosomal enzymes (Figure 7i, upper panel, lane 3). In cells expressing the GCase_Asn370Ser_ mutant, the 33 kDa Halo fragment is still generated (Figure 7i, upper panel, lane 4, Halo fragment, and Figure 7j). However, in cells expressing the GCase_Leu444Pro_ mutant, the 33 kDa Halo fragment is either absent or negligible (Figure 7i, upper panel, lane 6, Halo fragment, and Figure 7j).

Both the imaging (Figure 7a–e) and the biochemical assays (Figure 7i–j) performed in MEF cells (and in HEK293 cells, see below) recapitulate experiments performed in other cell lines and models (e.g., in fly models of GD and PD) [9] in demonstrating defective lysosomal delivery of the GCase_Leu444Pro_ protein, which is associated with severe disease phenotypes linked to *GBA1* variants.

#### 2.6.3. GT-02287 and GT-02329 Treatment Restores Lysosomal Transport of GCase_Leu444Pro_ in Cellulo

**Figure 8:**
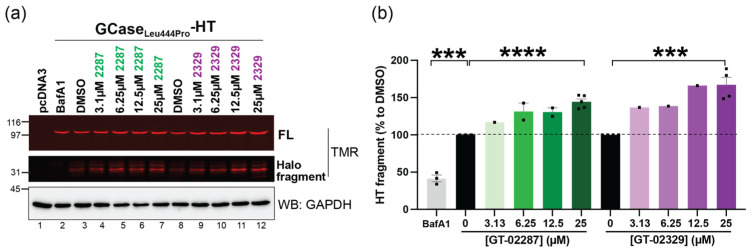
STAR compounds promote lysosomal delivery of the ER-retained variant GCase_Leu444Pro_: (**a**) TMR fluorescence of GCase_Leu444Pro_-HT was analyzed in HEK293 cells treated for 4 days with either DMSO or GT compounds (GT-02287 or GT-02329) at concentrations ranging from 3.1 µM to 25 µM. During the last 17 h of treatment, cells were incubated with 100 nM TMR. Control cells were treated with 50 nM BafA1 for 17 h (lane 2). After treatment, proteins were transferred onto a PVDF membrane and immunoblotted against GAPDH; (**b**) the Halo fragment was quantified, corrected for the GAPDH signal, and normalized to the DMSO control. The mean ± SEM is shown, with four independent experiments for 25 µM concentrations, two for 6.25 µM and 12.5 µM, and three for BafA1. Statistical analysis was performed using an unpaired *t*-test, with *** *p* < 0.001 and **** *p* < 0.0001. BafA1, bafilomycin A1; DMSO, dimethyl sulfoxide; GAPDH, glyceraldehyde 3-phosphate dehydrogenase; HEK293, human embryonic kidney 293 cells; PVDF, polyvinylidene fluoride; SEM, standard error of the mean; TMR, tetramethylrhodamine.

The performance of GT-02287 and GT-02329 was quantitatively assessed by monitoring the lysosomal generation of the Halo fragment in cells expressing the transport-deficient GCase_Leu444Pro_-HT chimera, using the previously validated quantitative biochemical assay. Cells expressing GCase_Leu444Pro_-HT were grown in the presence of 100 nM TMR and exposed for 4 days to increasing concentrations of GT-02287 (Figure 8a, lanes 4–7) or GT-02329 (Figure 8a, lanes 9–12). After treatment, the cells were detergent-solubilized, and TMR-labeled polypeptides in the PNS were visualized in gel using a 532 nm wavelength laser. The inhibition of lysosomal hydrolases with BafA1 prevents the generation of the Halo fragment (Figure 8a, lane 2 vs. lane 3; Figure 8b, BafA1 vs. dimethyl sulfoxide [DMSO]). The fluorescence intensity of the Halo fragment in mock-treated cells (Figure 8a, lanes 3 and 8; Figure 8b, DMSO) reflects the basal level of GCase_Leu444Pro_-HT delivered to lysosomal compartments, which is significantly lower than the levels observed for GCase and GCase_Asn370Ser_ (Figure 7i,j). Notably, exposure to GT-02287 (Figure 8a, lanes 4–7) or GT-02329 (Figure 8a, lanes 9–12) increases the generation of the Halo fragment in a dose-dependent manner (Figure 8b).

These results demonstrate that both GT-02287 and GT-02329 significantly enhance the transport of the GCase_Leu444Pro_ protein from the ER to the lysosome, its site of activity.

### 2.7. GT-02287 and GT-02329 Promote GCase_Leu444Pro_ Release from ER-Resident Chaperones and Inhibit Its Proteasomal Clearance

**Figure 9:**
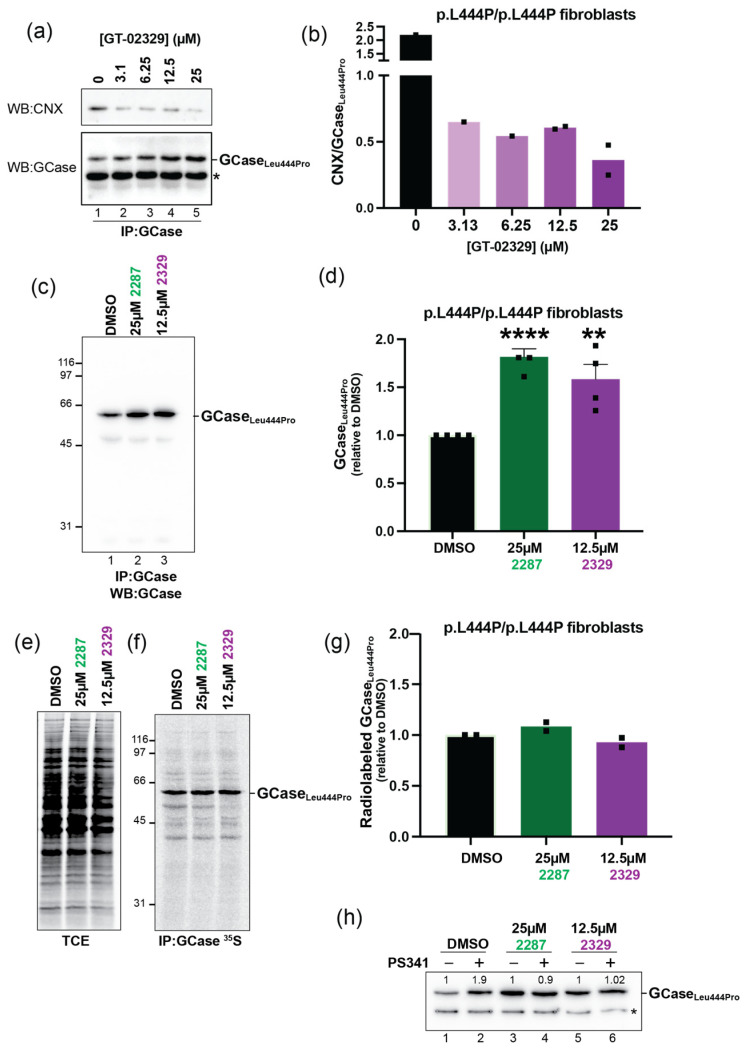
STAR compounds promote CNX release and prevent proteasomal degradation of GCase_Leu444Pro_: (**a**) co-immunoprecipitation of endogenous GCase_Leu444Pro_ (lower panel) with CNX (upper panel) was analyzed in patient fibroblasts (Coriell GM08760) treated for 4 days with DMSO or the compound GT-02329 at concentrations ranging from 3.1 µM to 25 µM. Protein content in the total cell extracts (TCEs) is shown. (*) indicates an unspecific band; (**b**) quantification of (**a**). The mean is shown, based on two independent experiments; (**c**) immunoisolated GCase_Leu444Pro_ from patient fibroblasts (Coriell GM10915) treated for 4 days with DMSO or compounds GT-02287; (**d**) quantification of (**c**). The mean ± SEM is shown, based on four independent experiments, using an unpaired *t*-test with ** *p* < 0.01 and **** *p* < 0.0001; (**e**) radiolabeled protein content in the TCE is shown; (**f**) radiolabeled GCase_Leu444Pro_ immunoisolated from patient fibroblasts (Coriell GM10915); (**g**) quantification of (**f**). The mean is shown, based on two independent experiments; (**h**) GCase_Leu444Pro_ levels in patient fibroblasts (Coriell GM08760) treated for 4 days with DMSO or compounds GT-02287 or GT-02329 and, for the last 3 h, with the proteasome inhibitor PS341. (*) indicates an unspecified band. CNX, calnexin; DMSO, dimethyl sulfoxide; TCE, total cell extracts; SEM, standard error of the mean; PS341, proteasome inhibitor (bortezomib).

The performance of STARs was evaluated in patient-derived primary human fibroblasts containing the homozygous 1448T > C variant in the *GBA1* gene, which corresponds to the Leu444Pro substitution. Exposure to increasing concentrations of GT-02329 for 4 days progressively reduced the co-precipitation of CNX (Figure 9a,b) and significantly increased the intracellular levels of endogenous GCase_Leu444Pro_ (Figure 9a, lanes 2–5). A similar increase in the intracellular GCase_Leu444Pro_ protein was observed in patient cells treated with GT-02287 (Figure 9c,d, lanes 2 and 3, respectively).

To determine whether the elevation of GCase_Leu444Pro_ in patient fibroblasts treated with STARs was due to enhanced synthesis of the endogenous polypeptide, cells were metabolically labeled for 10 min with 35S-methionine and -cysteine. Analysis of radiolabeled polypeptides (Figure 9e) and radiolabeled GCase_Leu444Pro_ (Figure 9f) in lysates from mock-treated fibroblasts (lanes 1) or fibroblasts exposed to GT-02287 or GT-02329 (lanes 2 and 3, respectively) revealed that STARs do not enhance total protein synthesis or the synthesis of endogenous GCase_Leu444Pro_. Instead, STARs substantially stabilize endogenous GCase_Leu444Pro_ by preventing its proteasomal clearance.

Consistently, in mock-treated cells, the GCase_Leu444Pro_ level is substantially increased when patient cells are exposed to PS341, a selective inhibitor of 26S proteasomes (Figure 9h, lanes 1 vs. 2) [39]. Treatment with GT-02287 (lane 3) or GT-02329 (lane 5) significantly raises the level of endogenous GCase_Leu444Pro_ (compare Figure 9h, lane 1 with lanes 3 and 5; Figure 9a–d). Incubation with PS341 does not further increase the GCase_Leu444Pro_ level, indicating that the turnover of GCase_Leu444Pro_ has already been substantially delayed by the STARs (Figure 9h, lanes 3 vs. 4 and 5 vs. 6).

### 2.8. GT-02287 and GT-02329 Alleviate ER Stress in p.L444p/p.L444P Patient-Derived Fibroblasts

**Figure 10:**
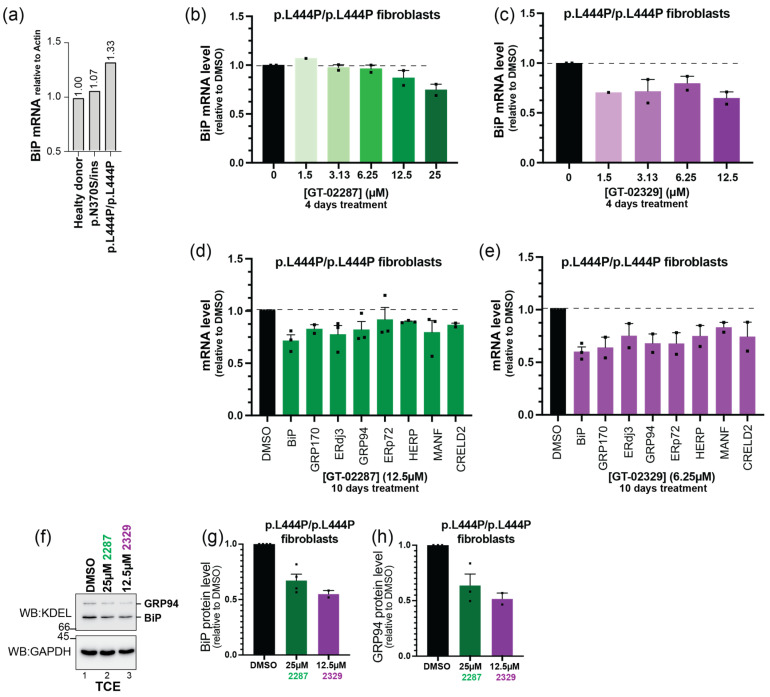
STAR compounds alleviate UPR in p.N370S/ins and p.L444P/p.L444P patient fibroblasts: (**a**) BiP mRNA levels determined by RT-PCR in fibroblasts from healthy donors (lane 1) and patient-derived fibroblasts (lanes 2 and 3); (**b**) BiP mRNA levels monitored by qPCR in GCase_Leu444Pro_ patient fibroblasts (Coriell GM08760) treated for 4 days with DMSO or the STAR compound GT-02287 at concentrations ranging from 1.5 µM to 25 µM; (**c**) same as (**b**), but for patient fibroblasts treated with GT-02329; the mean ± SEM is shown, based on two independent experiments; (**d**) mRNA levels of selected UPR markers monitored by qPCR in GCase_Leu444Pro_ patient fibroblasts (Coriell GM10915) treated for 10 days with DMSO or 12.5 µM GT-02287; the mean ± SEM is shown, based on two independent experiments for GRP170 and CRELD2, three for the other UPR markers; (**e**) same as (**d**), but for cells treated with 6.25 µM GT-02329; the mean ± SEM is shown, based on three independent experiments for BiP, two for the other UPR markers; (**f**) BiP and GRP94 protein levels monitored by WB analysis in GCase_Leu444Pro_ patient fibroblasts (Coriell GM08760) treated for 4 days with 25 µM GT-02287 and 12.5 µM GT-02329; (**g**,**h**) quantification of (**f**). The mean ± SEM is shown based on four independent experiments for BiP 25 μM 2287, three for GRP94 25 μM 2287, and two for 12.5 μM 2329. UPR, unfolded protein response; BiP, binding immunoglobulin protein; mRNA, messenger ribonucleic acid; RT-PCR, reverse transcription polymerase chain reaction; qPCR, quantitative polymerase chain reaction; DMSO, dimethyl sulfoxide; STAR, structurally targeted allosteric regulators; GRP94, glucose-regulated protein 94; WB, Western blot; SEM, standard error of the mean.

The engagement of ER-resident chaperones by folding-defective polypeptides induces low levels of UPR, characterized by the transcriptional and translational induction of a limited number of ER stress markers [40]. Persistent engagement of the ER chaperones BiP and CNX by the GCase_Leu444Pro_ mutant (Figure 10f) is reflected in the constitutively elevated levels of BiP transcripts in p.L444P/p.L444P mutant GD patient fibroblasts, as established by real-time quantitative polymerase chain reaction (qPCR) (Figure 10a). This indicates that p.L444P/p.L444P mutant GD patient fibroblasts experience higher basal levels of ER stress compared to fibroblasts from healthy individuals and from patients expressing the N370S mutant protein (Figure 10a). Notably, previous studies have reported elevated ER stress levels in mammalian and insect cells expressing mutant forms of GCase [10]. In contrast, our analyses show that p.N370S/ins patient-derived fibroblasts exhibit normal levels of BiP, consistent with normal ER homeostasis (Figure 10a).

The STAR compounds of GCase significantly reduce chaperone engagement and ER retention of GCase_Leu444Pro_ (Figure 10a,b), facilitating the delivery of the mutant polypeptide to the lysosomal compartment (Figure 8). This reduces the lysosomal accumulation of GCase substrates and has positive effects on cellular homeostasis. To determine whether this also alleviates the constitutive ER stress observed in patient cells, we monitored the transcript and protein levels of ER stress markers upregulated in cells expressing misfolded polypeptides [40].

Our experiments demonstrate that ER stress levels in patient fibroblasts exposed for 4 days to various concentrations of GT-02287 (Figure 10b) or GT-02329 (Figure 10c) are reduced by 20–40%, as shown by the quantification of BiP transcripts via real-time qPCR (Figure 10b,c) and the levels of transcripts of other ER stress markers (Figure 10d,e). More importantly, the reduction in ER stress is further supported by lower levels of BiP and GRP94 proteins in cells treated with the two STAR compounds of GCase (Figure 10f,h).

## 3. Discussion

This study highlights the therapeutic potential of two STARs, GT-02287 and GT-02329, designed to enhance the function of lysosomal GCase. We utilized the innovative SEE-Tx^®^ drug discovery platform to identify an allosteric, druggable binding site on GCase and developed these small-molecule PCs to bind outside the enzyme’s catalytic site. By stabilizing the enzyme, GT-02287 and GT-02329 increased GCase activity, enhanced lysosomal transport, and alleviated the cellular consequences of variants in the *GBA1* gene associated with GD and other GCase-related disorders.

Previous research has extensively documented the misfolding and retention of mutant GCase within the ER, where it is subjected to ERAD. Early studies [2,6] highlighted these mechanisms in tissue culture, while later studies [4,8] corroborated them in Drosophila models. PCs like ambroxol and arimoclomol have been investigated to alleviate these effects, with ambroxol improving trafficking and arimoclomol activating heat shock responses [9,12,13]. However, their stabilization of GCase without significantly enhancing activity underscores the need for innovative approaches like STAR compounds, which target both stability and enzymatic function.

The therapeutic potential of STAR compounds, such as GT-02287 and GT-02329, in PD and Lewy body disorders warrants a more detailed evaluation. PD pathogenesis involves both gain-of-function toxic α-synuclein aggregates and loss-of-function lysosomal dysfunction. While these compounds may enhance GCase activity and aid lysosomal clearance, effectively addressing loss-of-function defects, their ability to counteract α-synuclein accumulation remains uncertain [41]. Given PD’s multifactorial nature, further research is needed to establish whether targeting GCase alone can provide sufficient therapeutic benefit, especially in the context of gain-of-function contributions to pathology.

Our findings revealed distinct mechanistic insights into the cellular defects caused by GCase variants, particularly Leu444Pro and Asn370Ser. The Leu444Pro variant resulted in pronounced protein folding issues, characterized by persistent retention in the ER due to prolonged interaction with ER-resident chaperones such as BiP and CNX. This prolonged interaction activated a UPR, as previously shown in a fly model of GD [4], and delayed the transport of GCase to lysosomes. Conversely, the Asn370Ser variant exhibited milder retention and stress responses, correlating with its less severe clinical manifestations. These key differences underscore the challenges in restoring GCase function in patients with different variant profiles, particularly for variants causing severe folding defects like Leu444Pro.

The extended engagement of ER chaperones by folding-defective polypeptides eventually triggers a restricted UPR, characterized by the induction of a class of chaperones, including BiP, GRP70, ERdj3, GRP94, ERp72, HERP, MANF, and CRELD2, recently associated with a transcriptional/translational response to the intraluminal accumulation of folding-defective polypeptides [40,42]. Notably, GT-02287 and GT-02329 reduce polypeptide retention by the ER chaperone system and protect mutant forms of GCase from unwanted proteasomal degradation, indicating enhanced folding capacity. This has an immediate positive effect, as shown by reduced ER stress levels in patient-derived cell lines and enhanced delivery of mutant polypeptides to lysosomal compartments.

Treatment with GT-02287 and GT-02329 successfully targeted these biological challenges. The compounds demonstrated their efficacy by enhancing GCase activity and reducing the accumulation of toxic substrates such as GlcCer in fibroblasts derived from GD patients with various *GBA1* variants. For the Leu444Pro mutant, STAR treatment reduced ER retention, facilitated the release of mutant GCase from chaperones such as BiP and CNX and redirected the enzyme to lysosomes. Testing with patient-derived fibroblasts, the HaloTag GCase chimeras confirmed these effects, with the results showing increased lysosomal activity and substrate clearance after STAR treatment. The observed reduction in ER stress markers such as BiP and GRP94 further supports the conclusion that GT-02287 and GT-02329 mitigate cellular stress by enhancing proper protein folding and transport.

Detailed characterization of the compounds’ mechanisms through biochemical and imaging techniques provided additional evidence for their functionality. SPR experiments and competition assays with IFG confirmed that GT-02287 and GT-02329 selectively bind GCase at an allosteric site, distinct from the active site, ensuring that enzymatic activity was retained. This specific binding mechanism was supported by nanoDSF experiments, which demonstrated compound-induced stabilization of recombinant GCase. Furthermore, dose-dependent effects on GCase substrate clearance and lysosomal transport were validated by advanced imaging methods, including the LysoQuant deep-learning tool for cargo quantification [32], as well as by using HaloTag-based protein trafficking assays [18].

Perhaps most significantly, these findings suggest that GT-02287 and GT-02329 have the potential to address multiple GCase-related disorders, including PD and DLB, in addition to GD. Both PD and DLB exhibit pathways of lysosomal dysfunction and protein misfolding akin to those observed in GD. By enhancing the lysosomal delivery of mutant GCase and reducing downstream substrate accumulation, STAR compounds could contribute to mitigating neurodegeneration in these diseases. The ability of GT-02287 and GT-02329 to alleviate ER stress and improve cellular homeostasis in patient-derived fibroblasts further supports their potential as disease-modifying therapies across a broad spectrum of GCase-related pathologies.

Existing treatments for GCase-related disorders, such as ERT and SRT, are effective but have limitations, including high costs and potential adverse effects. In contrast, GT-02287 and GT-02329 offer a novel approach by enhancing GCase activity through allosteric modulation, potentially providing a more targeted and cost-effective therapeutic strategy. Unlike traditional PCs that may stabilize the enzyme but not necessarily enhance its activity, these STARs demonstrate a dual benefit by both stabilizing mutant GCase and increasing its enzymatic function. This unique mechanism of action positions GT-02287 and GT-02329 as promising candidates for addressing the unmet needs in the treatment of GD and other GCase-related disorders.

While our findings highlight the therapeutic potential of GT-02287 and GT-02329 for GCase-related disorders, several limitations of this study should be acknowledged. Notably, the experiments were conducted in cell-based models, and the efficacy of these compounds in vivo remains to be demonstrated. Additionally, due to sample or resource limitations, some experiments were conducted with duplicate measurements rather than the recommended triplicates, which may affect the robustness of the variability estimates. Animal studies are essential to validate the therapeutic effects on tissue distribution, lysosomal function, and overall pathology. Additionally, although these compounds were shown to stabilize and enhance GCase activity in patient-derived fibroblasts, the lack of clinical data limits conclusions about their potential effectiveness in patients with GCase-related disorders. Future research should focus on addressing these limitations through in vivo studies, clinical evaluations, and expanded testing in diverse biological systems.

Follow-up studies should explore the long-term effects of these compounds on lysosomal function, cellular stress, and neurodegenerative processes, particularly in animal models of GD and PD. Additionally, evaluating the pharmacokinetics, safety, and efficacy of GT-02287 and GT-02329 in clinical settings will be critical to determining their viability as therapeutic agents. However, the results of this study strongly validate the mechanism of action of these STAR compounds and reinforce their promise as novel treatment strategies for GCase-related disorders, paving the way for further therapeutic development.

## 4. Materials and Methods

### 4.1. Virtual Screening Using SEE-Tx^®^ Technology

The published three-dimensional (3D) structure of human GCase, refined to a resolution of 1.95 Å, was used (PDB ID: 2V3F). Molecular dynamics simulations in organic-aqueous solvent mixtures (MDmix v0.1, Computational Biology and Drug Design Group (CBDD), Barcelona, Spain) revealed a druggable cavity and identified key interaction sites, or binding hot spots, which were used as pharmacophoric restraints to guide docking and assess the binding site’s flexibility. A virtual library of approximately 5 million non-redundant chemical compounds from five vendors—Asinex (Moscow, Russia), Enamine (Kyiv, Ukraine), Life Chemicals (Kyiv, Ukraine), Princeton Biomolecular Research Inc. (Monmouth Junction, NJ, USA), and Specs (Zoetermeer, The Netherlands)—along with compounds from our GAIN DB library, was computationally evaluated using the rDock 2013.1 program (University of Barcelona, Barcelona, Spain). The evaluation employed a standard scoring function, pharmacophoric restraints, and a high-throughput protocol.

### 4.2. Binding Studies by SPR

Human full-length wild-type GCase protein (Cerezyme^®^, Genzyme, Naarden, Netherlands) was immobilized on a CM5 sensor chip (#29149603, Cytiva, Chicago, IL, USA) through standard amine coupling, using a protein concentration of 100 µg/mL. A nine-point, 2-fold serial dilution of GT-02287 or GT-02329 (starting from 100 µmol/L, prepared from a 10 mmol/L stock solution in DMSO) was analyzed at two pH levels: pH 7.4 (10 mmol/L HEPES, 5 mmol/L EDTA, 150 mmol/L NaCl, 0.01% Tween-20) and pH 5.0 (20 mmol/L Na phosphate, 2.7 mmol/L KCl, 137 mmol/L NaCl, 5 mmol/L tartrate, 0.01% Tween-20).

The SPR sensor included reference channels (empty, activated, and deactivated) for signal correction. Raw SPR data from the active channel were double-referenced by subtracting signals from the reference sensor surface and buffer-only controls, with additional corrections for DMSO signal mismatches. Binding affinities were calculated by fitting the SPR data to a four-parameter dose-response curve using GraphPad Prism 10.2.3 software.

To determine the mode of action of the compounds, competition experiments were conducted with IFG, a competitive inhibitor. IFG was included at saturating concentrations in the running buffer under both pH conditions (7.4 and 5.0). Competition experiments were performed as single measurements and repeated twice for reproducibility.

### 4.3. Binding Studies by nanoDSF

The stabilizing effects of GT-02287 and GT-02329 on GCase were assessed by nanoDSF. rhGCase protein (Cerezyme^®^, Genzyme, Naarden, Netherlands) was prepared at 0.1 mg/mL in two buffers: buffer 1 (10 mmol/L Citric Acid, 20 mmol/L Na_2_HPO_4_, pH 7.0) and buffer 2 (50 mmol/L Histidine, 150 mmol/L NaCl, pH 5.0). DMSO was added to a final concentration of 2%.

Samples were loaded into nanoDSF standard capillaries and analyzed using the Prometheus NT.48 instrument (NanoTemper Technologies, Munich, Germany). The thermal unfolding of GCase was monitored by tracking intrinsic fluorescence changes in tryptophan and tyrosine residues as the temperature increased from 20 °C to 95 °C at 2 °C/min. Each condition was run in duplicate at 25 μM and 100 μM compound concentrations. Melting temperatures (Tm) were determined from the fluorescence curves, and ΔTm shifts between compound-treated and control samples were calculated. The significance of Tm shifts was assessed using two criteria: an absolute ΔTm shift greater than 1.0 °C (instrumental criterion) and an absolute ΔTm standard deviation of 0.2 °C or less (statistical criterion).

### 4.4. GCase Biochemical and Competition Assay in Wild-Type Lysates

Lysates were prepared from wild-type fibroblasts in a lysis buffer containing 0.9% NaCl and 0.01% Triton-X100. The protein concentration was measured using a bicinchoninic acid (BCA) protein assay kit. To block 50% of GCase activity, lysates were pre-incubated with 40 µM CBE for 15 min, followed by a 15-min incubation with GT-02287 or GT-02329. Reactions were conducted using 5 mM 4-methylumbelliferyl-beta-D-glucopyranoside substrate in 0.1 M citrate-phosphate buffer (pH 5.6) at 37 °C for one hour. The reaction was stopped with 140 µL of 100 mM Glycine-NaOH buffer (pH 10.7). The fluorescence of released 4-methylumbelliferyl (4-MU) was measured on a Glomax^®^ Discover microplate reader (excitation 340 nm, emission 460 nm).

### 4.5. Cell Culture, Transient Transfection, and Use of Compounds

Mouse embryonic fibroblasts (MEFs) and Human Embryonic Kidney 293 (HEK293) cell lines were cultured at 37°C with 5% CO_2_ in Dulbecco’s Modified Eagle Medium (DMEM) high glucose (GlutaMAX™, Gibco, ThermoFischer Scientific, Reinach, Basel, Switzerland), supplemented with 10% Fetal Bovine Serum (FBS, Gibco, ThermoFischer Scientific, Reinach, Basel, Switzerland). Fibroblasts derived from GD patients were obtained from the Coriell Institute for Medical Research and the Telethon Network of Genetic Biobanks. The specific genotypes included were as follows: p.N370S/84GG (GD type I, Coriell GM00372), p.L444P/p.L444P (GD type II, Coriell GM08760), p.L444P/p.L444P (GD type III, Telethon 20526), p.L444P/p.L444P (GD type I, Coriell GM10915), p.L444P/p.F213I (GD type III, Telethon 21142), p.L444P/p.R496C (GD type III, Telethon 20624), and p.N188S/p.S107L (GD type III, Telethon 20843). Healthy fibroblasts (Coriell GM03377) were included as controls.

While the GM10915 cell line (p.L444P/p.L444P) is designated as type I by the Coriell Institute, it is important to note that the L444P/L444P genotype is most frequently associated with neuronopathic forms of Gaucher disease (types II or III). However, clinical studies have documented phenotypic variability among L444P homozygotes, including cases presenting as non-neuronopathic type I disease, underscoring the influence of genetic and environmental modifiers on disease expression.

For enzyme enhancement and substrate depletion studies, fibroblasts were maintained in DMEM supplemented with 10% heat-inactivated FBS and 1% penicillin-streptomycin at 37 °C under 5% CO_2_. For other cellular studies, patient fibroblasts were cultured in DMEM-GlutaMAX™ supplemented with 15% non-inactivated FBS under identical conditions.

Transient transfections were performed using JetPrime (Polypus) following the manufacturer’s protocol in DMEM supplemented with 10% FBS and non-essential amino acids (NEAA, Gibco). Pharmacological chaperones (GT-02287 and GT-02329; Gain Therapeutics) were dissolved in DMSO (Sigma) and applied at concentrations specified in the figure legends. The GCase inhibitor, CBE (Merck Millipore; Catalogue No. 234599), was used for enzyme inhibition studies, while the proteasome inhibitor PS341 (Bortezomib, LubioScience) was employed at a final concentration of 10 µM.

### 4.6. Plasmids and Cloning

Plasmids encoding GCase-HA (human influenza hemagglutinin), GCase-Halo N370S, and L444P mutant polypeptides within the pcDNA3.1(+) backbone, flanked by HindIII and XhoI restriction sites, were synthesized by GenScript. HA and HaloTag tags were transferred between plasmids through the restriction digestion of NotI and XhoI sites flanking the tags. Ligation was performed using T4 DNA ligase (NEB) at a 3:1 insert-to-vector ratio, following the manufacturer’s protocol. The ligated plasmids were amplified in JM109 bacteria (Promega) and purified using the GenElute™ HP Plasmid MidiPrep Kit (Sigma).

### 4.7. CLSM

MEFs were seeded on alcian blue-treated (Sigma-Aldrich Chemie GmbH, Buchs, Sankt Gallen, Switzerland) glass coverslips (VWR International GmbH, Dietikon, Switzerland) and transiently transfected using JetPrime (Polyplus, VWR International GmbH, Dietikon, Switzerland) in DMEM 10% FBS supplemented with non-essential amino acids (NEAAs; Gibco, ThermoFischer Scientific, Schlieren, Zurich, Switzerland), following the manufacturer’s protocol. Thirty-two hours post-transfection, BafA1 (Calbiochem, Merck Millipore, Schaffhausen, Switzerland) was added to the cell medium at a final concentration of 50 nM for 17 h. Cells expressing HaloTag-fusion proteins were supplemented with 100 nM TMR HaloTag ligand (Promega AG, Dübendorf, Switzerland).

After treatment, MEFs were fixed at room temperature (RT) for 20 min in 3.7% formaldehyde (FA, *v*/*v*) prepared in phosphate-buffered saline (PBS). Coverslips were incubated for 20 min in permeabilization solution (PS) containing 10 mM HEPES, 15 mM glycine, 10% goat serum (*v*/*v*), and 0.05% saponin (*w*/*v*). Following permeabilization, primary antibodies, diluted 1:100 in PS (unless noted otherwise; see Table A1), were applied for 120 min. Cells were washed three times with PS, and Alexa Fluor-conjugated secondary antibodies diluted 1:300 in PS were subsequently applied for 45 min. Post-incubation, cells were washed three times in PS and once with deionized water, then mounted using Vectashield (Vector Laboratories Inc., Newark, CA, USA) supplemented with 4′,6-diamidino-2-phenylindole (DAPI).

Coverslips were imaged using a Leica TCS SP5 microscope (Leica Microsystems GmbH, Wetzlar, Germany) equipped with a Leica HCX PL APO lambda blue 63.0 × 1.40 oil objective. Image acquisition utilized Leica LAS X software 4.5.0.025531 (Leica Microsystems GmbH, Wetzlar, Germany), with excitation provided by 488-, 561-, and 633-nm lasers. Fluorescence emissions were collected using the following ranges: 504–587 nm (AlexaFluor488), 557–663 nm (TMR), and 658–750 nm (AlexaFluor646), with the pinhole set to 1 AU.

The accumulation of GCase variants within LAMP1-positive endolysosomes was quantified using the LysoQuant plugin in ImageJ software 2.16.0/1.54p (U.S. National Institutes of Health, Bethesda, MD, USA). Image post-processing was performed in Adobe Photoshop.

### 4.8. SDS-PAGE, HaloTag Cleavage Assay, Co-Immunoprecipitation, and Western Blot

HEK293 cells or patient fibroblasts were washed with ice-cold 1× PBS containing 20 mM N-ethylmaleimide (NEM), then lysed in either RIPA buffer (1% Triton X-100 [*v*/*v*], 0.1% SDS [*w*/*v*], 0.5% sodium deoxycholate in HEPES-buffered saline [HBS], pH 7.4) or 2% CHAPS buffer (in HBS, pH 7.4) supplemented with 20 mM NEM and protease inhibitors (1 mM phenylmethylsulfonyl fluoride [PMSF], 16.5 mM Chymostatin, 23.4 mM Leupeptin, 16.6 mM Antipain, 14.6 mM Pepstatin). The lysates were incubated on ice for 20 min. To isolate the post-nuclear supernatant (PNS), lysates were centrifuged at 10,600× *g*, 4 °C, for 10 min.

The PNS was denatured and reduced by adding 100 mM dithiothreitol (DTT; F. Hoffmann-La Roche AG, Basel, Switzerland) and heating at 95 °C for 5 min. Samples were resolved on 12% acrylamide SDS-PAGE gels. Lysates containing GCase-HT variants labeled with the fluorescent TMR Halo ligand (Promega AG, Dübendorf, Switzerland) were imaged in gel using a Amersham Typhoon scanner (Cytiva, Marlborough, MA, USA) with a 532 nm laser. Band intensities of TMR signals were quantified with ImageJ software 2.16.0/1.54p (U.S. National Institutes of Health, Bethesda, MD, USA). Total protein content was assessed by staining the polyacrylamide gel with 0.25% Brilliant Blue R 250 (Sigma-Aldrich Chemie GmbH, Buchs, Sankt Gallen, Switzerland) in a solution of 50% methanol (*v*/*v*) and 10% acetic acid (*v*/*v*) for 20 min at room temperature (RT), followed by destaining in a solution of 20% methanol (*v*/*v*) and 7.5% acetic acid (*v*/*v*). Protein bands were captured using a Fusion FX7 system (Vilber) equipped with a transilluminator and analyzed using ImageJ software 2.16.0/1.54p (U.S. National Institutes of Health, Bethesda, MD, USA).

For immunoprecipitation, lysates from cells treated with DMSO or GT compounds were diluted with lysis buffer and incubated with specific bait antibodies and protein A-conjugated beads (1:10 [*w*/*v*], swollen in PBS) at 4 °C for 4 h. Beads were washed three times with 0.5% Triton X-100 (*v*/*v*) in HBS (pH 7.4) and centrifuged between washes. Denatured samples from the beads were heated for 5 min at 95 °C and analyzed by SDS-PAGE.

For Western blotting (WB), proteins were transferred from polyacrylamide gels to polyvinylidene fluoride (PVDF) membranes using the TransBlot Turbo device (Bio-Rad Laboratories AG, Cressier, Fribourg, Switzerland). The PVDF membranes were blocked for 10 min with 10% milk (BioRad, *w*/*v*) in Tris-buffered saline and 0.1% Tween 20 (TBS-T), briefly rinsed in TBS-T, and incubated overnight at 4 °C with primary antibodies (see Appendix A Table A1) under agitation. After washing out the primary antibodies with TBS-T, membranes were incubated with HRP-conjugated secondary antibodies or protein A (see Appendix A Table A1) for 45 min at RT with shaking. Protein bands were detected using the Fusion FX7 chemiluminescence detection system (Vilber, Collégien (Marne-la-Vallée), France) and the WesternBright™ Quantum system (Advansta Inc., San Jose, CA, USA), according to the manufacturer’s instructions. Protein band intensities were quantified with ImageJ software 2.16.0/1.54p (U.S. National Institutes of Health, Bethesda, MD, USA).

### 4.9. Radioactive Metabolic Labelling

Four days after treatment with DMSO or GT compounds, patient fibroblasts were incubated for 4 h with 0.2 mCi [35S] methionine/cysteine mix. The pulse was followed by a 10 min chase with DMEM supplemented with 5 mM cold methionine and cysteine. Cells were lysed in RIPA buffer, and radiolabeled proteins were visualized using an Amersham Typhoon scanner (Cytiva, Marlborough, MA, USA). The intensity of radioactive bands was quantified using ImageJ software 2.16.0/1.54p (U.S. National Institutes of Health, Bethesda, MD, USA).

### 4.10. RNA Extraction and Reverse Transcription-Quantitative Polymerase Chain Reaction

Patient fibroblasts treated with DMSO or GT compounds were detached with trypsin and then centrifuged at 450× *g* for 5 min. Total RNA was extracted using the GenElute™ Mammalian Total RNA Miniprep Kit (Sigma-Aldrich International GmbH (Merck), Buchs, Sankt Gallen, Switzerland) following the manufacturer’s protocol. The isolated mRNA was reverse transcribed into cDNA using BioTool™ 2× SYBR Green qPCR Master Mix (BioTool AG, Kirchberg, Bern, Switzerland) according to the manufacturer’s instructions.

For quantitative polymerase chain reactions (qPCRs), the master mix was combined with qPCR primers (final concentration 10 µM, obtained from Microsynth AG, Balgach, Switzerland) on a reading plate (see Appendix A Table A2). qPCRs were performed using the QuantStudio™ 3 Real-Time PCR System (Applied Biosystems, ThermoFischer scientific, Schlieren, Zurich, Switzerland). Data analysis was carried out with QuantStudio™ Design & Analysis Software v1.5.5 (Applied Biosystems, ThermoFischer scientific, Schlieren, Zurich, Switzerland).

### 4.11. GCase Enzyme Enhancement Assay

Patient-derived fibroblasts were seeded at 5000 cells per well in 96-well cell culture plates containing DMEM supplemented with 10% FBS, 1% P/S (Thermo Fisher Scientific, Waltham, MA, USA). Cells were incubated at 37 °C (Nüve EC160, Nüve Sanayi Malzemeleri İmalat ve Ticaret A.Ş., Ankara, Turkey), 5% CO_2_ overnight for attachment. The following day, cells were treated with or without the indicated compounds at specified concentrations for 4 days. After incubation, cells were washed with PBS and incubated with 5 mM 4-methylumbelliferyl-β-D-glucopyranoside (Apollo Scientific Ltd., Denton, Manchester, UK) in 0.1 M acetate buffer, pH 4, for 1 h at 37 °C. The reaction was stopped by adding 200 µL of 100 mM glycine-NaOH buffer, pH 10.7. The released 4-MU was quantified using a GloMax^®^ Discover microplate reader, with excitation at 340 nm and emission at 460 nm.

### 4.12. GRP94 Quantification in Patient-Derived Fibroblasts

Patient-derived fibroblasts were seeded at 750,000 cells in 75 cm^2^ flasks containing DMEM supplemented with 10% FBS and 1% P/S. The next day, cells were treated with compounds at specified concentrations. On day 3 and day 7, the medium was replaced with fresh media containing the compound. On day 10, the supernatant was discarded, fibroblasts were trypsinized, and cell media were added to the detached cells before collection in tubes. The samples were centrifuged with cold PBS, and the pellets were stored at −80 °C.

GlcCer quantification was conducted as follows: Cell culture pellets were transferred into Eppendorf tubes, weighed, and stored in an ice-water bath. Deionized water (1:10, weight/volume) and deuterium-labeled GlcCer-D5 (internal standard) were added. The pellets were resuspended in water using an ultrasonic homogenizer VCX130 (Sonics & Materials Inc., Newtown, CT, USA). Protein content was quantified from 10 µL of resuspended samples using a Pierce Rapid Gold BCA protein assay (Thermo Fisher Scientific, Waltham, MA, USA) and a UV/VIS plate reader (Labrox Oy, Turku, Finland).

From the remaining sample, 70 µL was used for GlcCer extraction via liquid–liquid extraction. The quantification of glycosphingolipids was performed using a UHPLC-MS/MS system consisting of a Xevo TQ-S micro triple quadrupole mass spectrometer and an ACQUITY UPLC system (Waters Micromass). The electrospray ionization (ESI) source operated in positive mode with the following parameters: capillary voltage of 4500 V, sample cone voltage of 30 V, source temperature of 150 °C, desolvation temperature of 500 °C, and nebulizer gas flow of 1000 L/hr. Acquisition parameters included centroid MRM scans of parent and daughter ions with 0.1-s dwell time.

The HALO HILIC column (2.1 × 150 mm, 2 μm particle size, Advanced Materials Technology, Wilmington, DE, USA) was used for the chiral separation of GlcCer from GalCer at +35 °C. The mobile phase consisted of 95% ACN, 2.5% MeOH, 2.5% water containing 5 mM ammonium formate, and 0.5% formic acid, with a flow rate of 0.240 mL/min. GlcCer levels in cell pellets were expressed as ng/mg protein.

Data analysis was performed using GraphPad Prism 5.0 Software, with values shown as mean ± SD (standard deviation) from three wells per condition. Statistical differences from untreated GCase_Leu444Pro_ samples were determined by one-way analysis of variance (ANOVA) followed by Dunnett’s Multiple Comparison Test. Asterisks indicate statistically significant values from untreated GCase_Leu444Pro_ **** *p* ≤ 0.0001 and *** *p* ≤ 0.001.

### 4.13. Statistical Analyses

For Figure 2, Figure 3, Figure 4 and Figure 5, data analysis was performed using GraphPad Prism 5.0 Software. For Figure 5, Figure 6, Figure 7 and Figure 8, statistical comparisons and graphical plots were performed in GraphPad Prism 10.4.2 (GraphPad Software Inc., Boston, MA, USA). An ordinary one-way ANOVA Dunnett’s multiple comparisons test was used to assess statistical significance (for Figure 6a,b). An adjusted *p*-value < 0.05 (for one-way ANOVA with Dunnett’s multiple comparison test) was considered statistically significant: *** *p* < 0.001 and **** *p* < 0.0001. For Figure 7c,j, Figure 8b and Figure 9d,g, an unpaired *t*-test was used to assess statistical significance. An exact *p*-value < 0.05 was considered significant; ns is not significant, * *p* < 0.05, ** *p* < 0.01, *** *p* < 0.001, and **** *p* < 0.0001. All replicates represent biological replicates, and for all statistical comparisons, the number of repetitions and the number of cells analyzed (n, for LysoQuant analyses) are indicated in the figure legends.

## 5. Conclusions

This study demonstrates the efficacy of STARs in enhancing GCase activity, an enzyme whose dysfunction is linked to GD and PD. The compounds GT-02287 and GT-02329, developed using the SEE-Tx platform, show significant potential by binding to an allosteric site on GCase, thereby increasing its stability and enzymatic activity. These findings introduce a novel therapeutic strategy for addressing the biochemical defects underlying these diseases.

The research highlights the potential of STARs to improve GCase activity in cells derived from patients with GD, while reducing the accumulation of toxic substrates. This approach offers a promising avenue for treating GD and potentially PD by targeting the root cause of GCase dysfunction.

The relevance of these findings to ongoing research is substantial. They provide a foundation for further investigation into the clinical application of STARs, which could lead to new therapeutic options for patients with GCase-related disorders. Future studies will be crucial to translating these preclinical results into clinical trials, focusing on safety, efficacy, and potential synergies with existing treatments. Overall, this research makes a significant contribution to the development of innovative therapies aimed at improving outcomes for individuals affected by these debilitating diseases.

## Figures and Tables

**Table 1:** SPR direct binding affinities (measured at pH 7.4 and 5.0) and competition data with IFG.

	pH 7.4		pH 5.0		Competition with IFG
Compound	KD (µM)	KD (µM) + IFG	KD (µM)	KD (µM) + IFG	
IFG (control)	0.03–0.06	-	0.10–0.11	-	-
GT-02287	17–24	27–30	50–150	55–64	No
GT-02329	>90	>90	39–40	30–31	No

K_D_, dissociation constant. IFG, isofagomine.

**Table 2:** Thermal stability effects on rhGCase determined by nanoDSF at pH 7.0 and 5.0.

Sample	ΔTm at pH 7.0 and 25 µM (°C)	ΔTm at pH 7.0 and 100 µM (°C)	ΔTm at pH 5.0 and 100 µM (°C)
GT-02287	1.3 ± 0.1	1.5 ± 0.0	0.0 ± 0.0
GT-02329	1.0 ± 0.1	2.4 ± 0.3	1.2 ± 0.0
IFG (control)	9.9 ± 0.2	11.5 ± 0.0	3.0 ± 0.0

The table shows the difference in melting temperature (ΔTm) relative to recombinant human glucosylceramidase beta 1 (rhGCase) in the presence of the two compounds (GT-02287 and GT-02329) at 25 μM and 100 μM. Values represent the mean ± SD of two independent experiments. IFG, isofagomine; SD, standard deviation.

**Figure A1:**
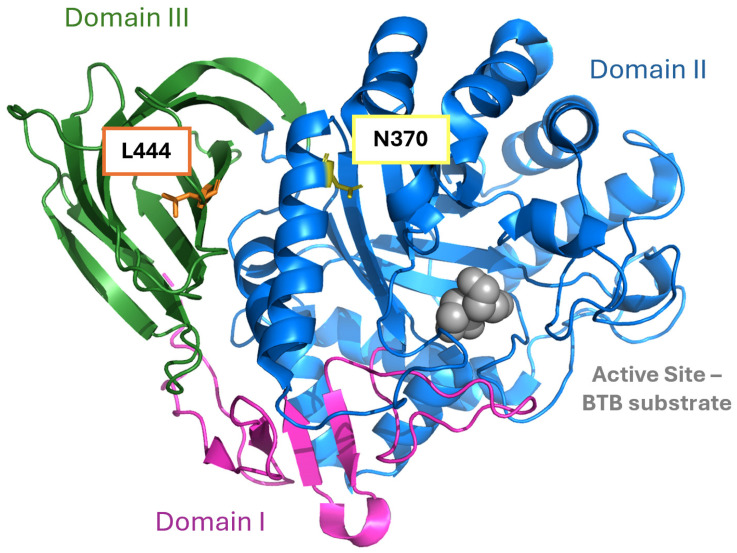
Structural localization of L444 and N370 amino acid residues in GCase. The molecular model shows the positions of L444 (orange sticks, Domain III) and N370 (yellow sticks, Domain II) relative to the enzyme’s active site (grey spheres, BTB substrate). Both residues are located distal to the catalytic pocket, supporting their involvement in allosteric regulation. Domain I (magenta), Domain II (blue), and Domain III (green) are depicted in ribbon representation. The structure was modeled using wild-type GCase (PDB 2V3F).

## Data Availability

Most data associated with this study are available in the main text or the Appendix A. Additional data and materials that support the findings of this study are available from the corresponding author upon reasonable request, with the following exceptions: The chemical structure of the compounds and the precise location of the allosteric binding site are not disclosed currently due to their proprietary nature and ongoing clinical development. This information may be made available once it enters the public domain or upon completion of the relevant patent applications. These restrictions are in place to protect the integrity of the ongoing drug development process and to comply with confidentiality agreements. We are committed to scientific transparency and will work with qualified researchers to provide as much information as possible without compromising the clinical development of these potential therapeutic agents.

## Abbreviations

The following abbreviations are used in this manuscript:

ANOVA	Analysis of variance
BafA1	Bafilomycin A1
BiP	Binding immunoglobulin protein
CBE	Conduritol-β-epoxide
CNX	Calnexin
CLSM	Confocal laser scanning microscopy
CRELD2	Cysteine-rich with EGF-like domains 2
cDNA	Complementary DNA
CNS	Central nervous system
DMEM	Dulbecco’s modified eagle medium
DMSO	Dimethyl sulfoxide
DNA	Deoxyribonucleic acid
DLB	Dementia with Lewy Bodies
ΔTm	Change in melting temperature
EDTA	Ethylenediaminetetraacetic acid
ER	Endoplasmic reticulum
ERp72	Protein disulfide isomerase A4
ESI	Electrospray ionization
FBS	Fetal bovine serum
GD	Gaucher Disease
GCase	Glucocerebrosidase
GlcCer	Glucosylceramide
GRP94	Glucose-regulated protein 94
HA	Hemagglutinin tag
HEK293	Human embryonic kidney 293 cells
HERP	Homocysteine-induced ER protein
HT	HaloTag
K_D_	Dissociation constant
LAMP1	Lysosomal-associated membrane protein 1
LysoQuant	Deep-learning fluorescence imaging tool
MEF	Mouse embryonic fibroblast
mRNA	Messenger RNA
NA	Not applicable
nanoDSF	Nano differential scanning fluorimetry
PDB	Protein Data Bank
PD	Parkinson’s Disease
PS341	Bortezomib
qPCR	Quantitative polymerase chain reaction
rhGCase	Recombinant human glucocerebrosidase
SD	Standard deviation
SEE-Tx^®^	Site-directed enzyme enhancement therapy
SEM	Standard error of the mean
SDS-PAGE	Sodium dodecyl sulfate-polyacrylamide gel electrophoresis
SPR	Surface plasmon resonance
STAR	Structurally targeted allosteric regulator
TMR	Tetramethylrhodamine
UPR	Unfolded protein response
WB	Western blot

## Appendix A

**Table A1:** Antibodies used in the study.

Antibody	Manufacturer	Catalog Number	Dilution
Rat anti-LAMP1	DSHB	1D4B	1:50 (CLSM)
Rabbit anti-HA	Sigma	H6908	1:100 (CLSM) 1:3000 (IB)
Mouse anti-KDEL	Stressgen	SPA-827	1:1000 (IB)
Rabbit anti-CNX	Kind gift from A. Helenius	Not applicable	1:3000 (IB)
Mouse anti-GAPDH	Merck	MAB374	1:30000 (IB)
Rabbit anti-GCase	Sigma	G4171	1:500 (IB)
Mouse anti-GCase	Abnova	H00002629-M01	1:500 (IB)
Protein A HRP-conjugated	Invitrogen	101023	1:20,000 (IB)
Goat anti-mouse HRP-conjugated	Southern Biotech	1031-05	1:20,000 (IB)
Goat anti-rabbit AlexaFluor488-conjugated	Thermo Fisher Scientific	A-21206	1:300 (CLSM)
Goat anti-rat AlexaFluor647-conjugated	Thermo Fisher Scientific	A-21247	1:300 (CLSM)

**Table A2:** Primers for quantitative polymerase chain reactions (qPCR).

Transcript	Forward Primer (5′-3′)	Reverse Primer (5′-3′)
BiP	GAGTTCTTCAATGGCAAGGA	CCAGTCAGATCAAATGTACCC
GRP170	GCAGACCTGTTGGCACTG	TCACGATCACCGGTGTTT
ERdj3	GGTGATTGCCGGACGAGATT	GGATCATCAGGGTTCCGGTC
GRP94	CTGGGTCAAGCAGAAAGGAG	TGCCAGACCATCCATACTGA
ERp72	ATCGGGGTCTTTAAGGGGGA	CTCTCAGGTTGTTAGCGGCA
HERP	CCGGTTACACACCCTATGGG	TGAGGAGCAGCATTCTGATTG
MANF	ATCGGTTGTGCTACTATA	CTCGGAGCTTCTCAGGT
CRELD2	ACAGCCTGTGACGAGTCCTGC	CTCGTCCACATCCACACAGGCG
GCase	TGCTGCTCTCAACATCCTTGCC	TAGGTGCGGATGGAGAAGTCAC

Surface plasmon resonance (SPR) competition experiments with IFG confirmed Gain Therapeutic GT-02287 and GT-02329 compounds bind to Cerezyme at both pH 7.4 and pH 5.0. Notably, these compounds do not bind to the enzyme’s active site but rather to a distinct pocket.
